# Video-assisted informed consent in cardiac imaging: influence on patient experience during CT—the randomized controlled VAICICI Trial

**DOI:** 10.1007/s00330-025-11741-8

**Published:** 2025-06-27

**Authors:** Robin F. Gohmann, Sophia M. Mettke, Christian Krieghoff, Christian Lücke, Fyn Kaiser, Patrick Seitz, Steffen Desch, Jonas Steglich, Alexander Bäuerle, Benedikt Schaarschmidt, Holger Thiele, Elfriede Strotdrees, Maria Buske, Michael A. Borger, Mohamed Abdel-Wahab, Matthias Horn, Matthias Gutberlet

**Affiliations:** 1 Department of Diagnostic and Interventional Radiology, Heart Center Leipzig, Leipzig, Germany; 2 Medical Faculty, University of Leipzig, Leipzig, Germany; 3 Department of Cardiology, Heart Center Leipzig at University of Leipzig, Leipzig, Germany; 4 Clinic for Psychosomatic Medicine and Psychotherapy, LVR-University Hospital, University of Duisburg-Essen, Essen, Germany; 5 Institute of Diagnostic and Interventional Radiology and Neuroradiology, University Hospital Essen, Essen, Germany; 6 Department of Cardiac Surgery, Heart Center Leipzig at University of Leipzig, Leipzig, Germany; 7 Institute for Medical Informatics, Statistics and Epidemiology (IMISE), University of Leipzig, Leipzig, Germany

**Keywords:** Informed consent process, Patient satisfaction, Patient education as topic, Educational technology, Computed tomography angiography

## Abstract

**Objective:**

Patient compliance and comfort during cardiac computed tomography (CT) are crucial for optimal imaging outcomes. Insufficient understanding or heightened anxiety can negatively impact patient experience and potentially image quality. This study evaluates the effectiveness of video-assisted informed consent in cardiac imaging (VAICICI) in improving patient satisfaction, preparedness, and anxiety during cardiac CT.

**Materials and methods:**

In this randomized controlled trial, 205 patients scheduled for cardiac CT were assigned to Video I (*n* = 67), Video II (*n* = 69), or Control (*n* = 69). Video I featured an instructional video incorporating visuals, narration, and subtitles. Video II included audio and subtitles only. All groups received a standard physician consultation. Experiences were assessed before and after cardiac CT using patient-reported outcome measures. Statistical analyses included group comparisons and multivariate analysis of anamnestic and demographic factors.

**Results:**

Video I and II significantly improved patient satisfaction and understanding compared to the Control (*p* < 0.05). Patients with Video II placed more importance on the informed consent form for understanding than the Control (*p* = 0.02). Satisfaction was higher in both video groups (*p* = 0.02), with no significant difference between them. Anxiety and nervousness levels were low and similar across groups, but notably higher among women (*p* = 0.008) and patients with suspected coronary artery disease (*p* = 0.04). Age, prior CT/MRI, and eight other baseline demographics partially predicted responses.

**Conclusion:**

VAICICI improves patient satisfaction and understanding of the cardiac CT examination, with only slight variations in effectiveness across demographics, suggesting it is a valuable addition to the informed consent process. No reduction in anxiety was observed.

**Key Points:**

***Question*** *Can video-assisted informed consent improve patients’ understanding and reduce anxiety associated with cardiac CT examinations?*

***Findings*** *Video-assisted informed consent improves patient satisfaction and understanding of the cardiac CT examination, with both video formats significantly outperforming the standard consent*.

***Clinical relevance*** *Video-assisted informed consent improves patient satisfaction and understanding of cardiac CT, with only slight variation in effectiveness across baseline demographics, suggesting it to be of value in the consent process and promoting patient-centered imaging practices*.

**Graphical Abstract:**

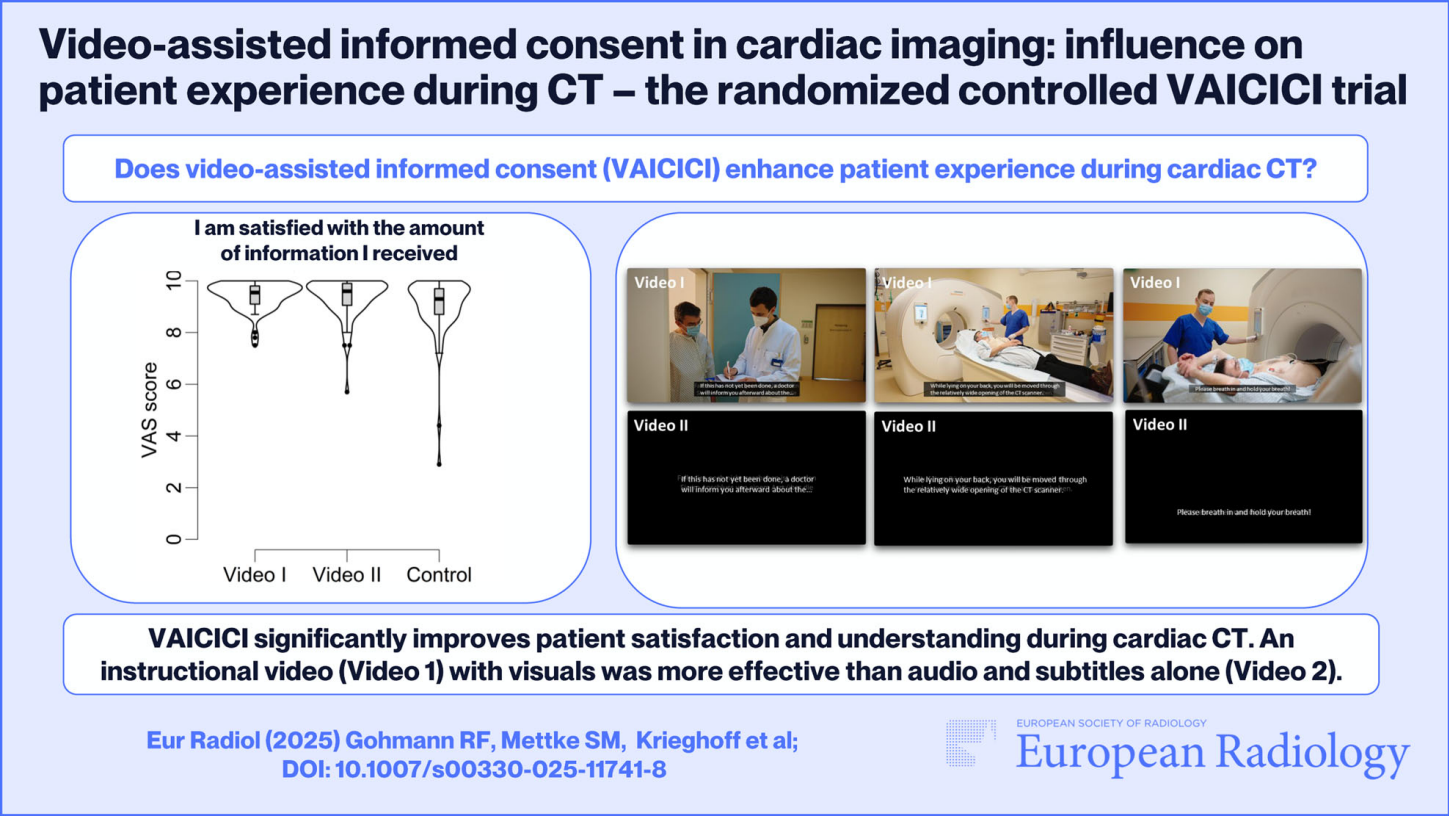

## Introduction

Cardiac computed tomography (CT) is a sophisticated and technically demanding radiological examination. Despite its complexity, coronary CT angiography (cCTA) serves as a non-invasive diagnostic tool for the assessment of coronary artery disease (CAD) and is the first-line imaging test in the workup of chronic coronary syndrome [1, 2]. Additionally, cCTA can be incorporated into many pre-operative CT examinations and pre-interventional planning for transcatheter aortic valve implantation (TAVI) [3, 4], without necessarily increasing the required amount of contrast medium [5]. With a low and steady heart rate, cCTA may even be incorporated without any additional radiation dose [6–8].

To achieve motion artifact-free cardiac CT images of the entire heart, patients’ compliance and calmness are of great importance. However, this can be challenging because patients often experience heightened anxiety before and during a cCTA examination [9, 10] due to factors like the unfamiliar examination environment with an intimidatingly appearing scanner and the unusual sensation during contrast medium injection. Several studies have shown that anxiety may reduce compliance during various examinations or procedures [9–12]. Somewhat contradictory, a smaller study with limited statistical power reported no significant effect of heightened anxiety on the image quality during cCTA [10].

Video-assisted informed consent (VAIC) has been shown to reduce anxiety and improve understanding in various procedures, including coronary angiography and percutaneous coronary intervention [11, 13–17]. However, VAIC’s application in cardiac CT, a diagnostic modality with unique periprocedural requirements posing additional challenges for patient compliance, remains underexplored, with the available studies assessing anxiety only before or after the examination, using non-standardized assessment instruments or having small sample sizes [10, 18].

The VAICICI trial addresses this gap as the first randomized controlled study to evaluate VAIC in cardiac imaging. Its three-arm design—comparing an instructional video, a minimalist video, and standard physician consultation—provides unique insights into how audiovisual information influences patient satisfaction, preparedness, and anxiety. Additionally, this trial explores the impact of a variety of baseline demographics on patients’ examination experiences.

## Materials and methods

### Study design

Between January and December 2023, patients scheduled for a cardiac CT examination at the Department of Diagnostic and Interventional Radiology, Heart Center Leipzig, Germany, were invited to join the prospective randomized controlled three-armed VAICICI trial. Due to the exploratory nature of the study, no specific sample size calculation was performed [19].

Inclusion criteria were: age ≥ 18 years, fluency in German, and competency to provide informed consent. Exclusion criteria were: pharmaceutically treated psychiatric illnesses, uncorrected visual or auditory impairments preventing the patient from reading the questionnaire or viewing a video.

The study complied with the Declaration of Helsinki (Medical Association 2013) and was approved by the local ethics committee (reference number: 172/22-ek). Written informed consent was obtained from all participants.

### Study setup

Following recruitment, patients were randomized into three groups (Video I, Video II, Control). To balance group sizes, block randomization with a 1:1:1 ratio in blocks of 30 was used. Randomization was conducted by a study physician. To ensure the imaging process remained unbiased, the technicians acquiring the CT examinations were blinded to the randomization and group allocation.

All patients received a physician consult with an interview and a two-part questionnaire. Part I of the questionnaire was to be answered after the standard physician interview with written informed consent. Part II was to be filled out after the imaging examination.

**Fig. 1:**
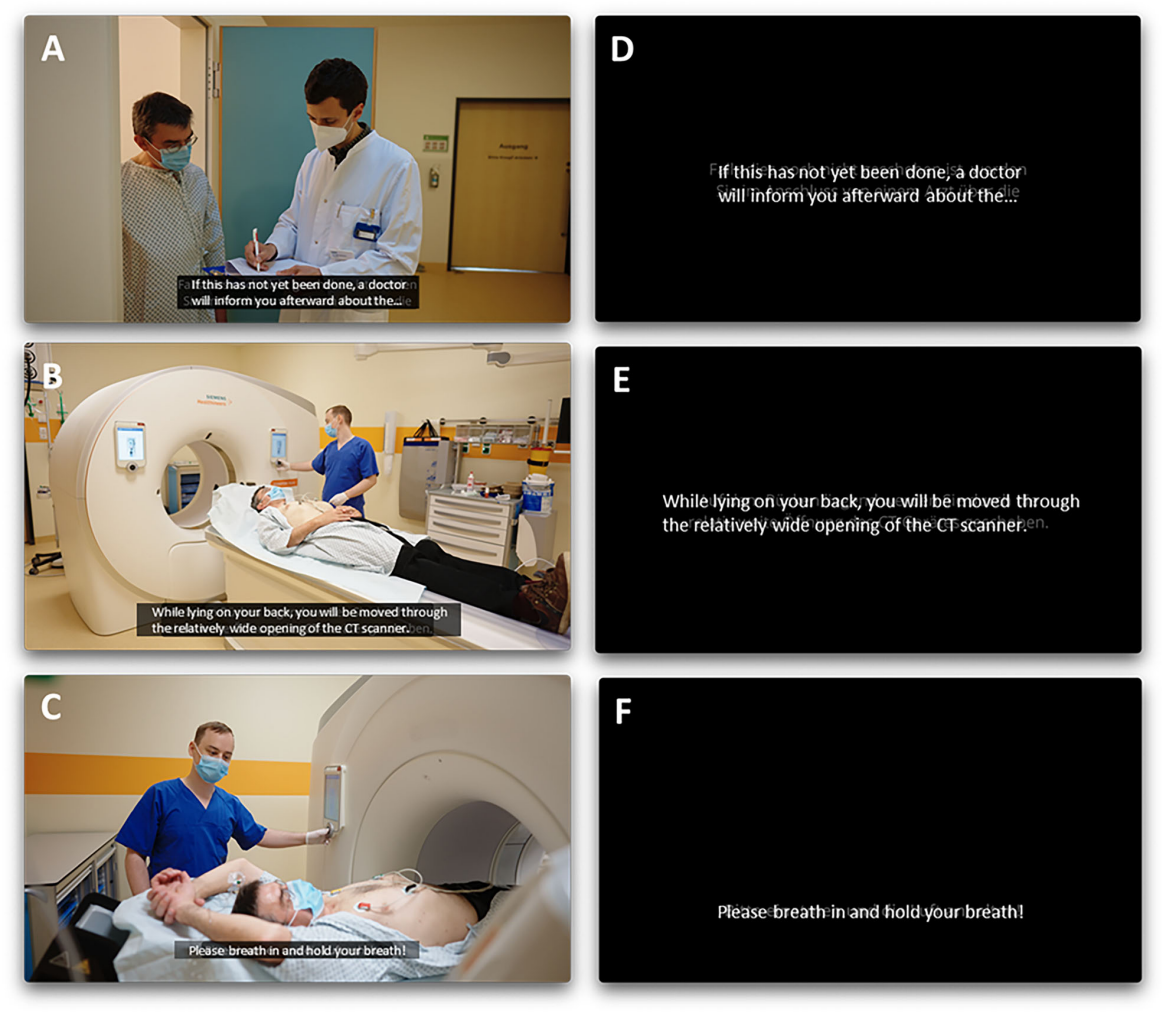
Screen captions of Video I and Video II: Screen captions from Video I (instructional video; **A**–**C**) and Video II (minimal video; **D**–**F**). Video I (**A**–**C**) depicts and narrates a patient’s journey from entering the hospital through key steps, including registration (not shown here), the physician encounter with informed consent (**A**), the examination process with table movement (**B**, **C**), and contrast injection and breath-hold instructions, supported by an animation (not shown). Video II (**D**–**F**) is identical in length and content to Video I, but is limited to audio and subtitles

Before the physician interview, patients in group Video I viewed an instructional video, and patients in Video II viewed an explanatory audio accompanied by subtitles without supporting visuals (Fig. 1). The Control received only the standard physician interview and no video. The standard physician interview was conducted by a staff physician and lasted 2–10 min. Its duration depended on the patient’s prior CT experience, specific indications (e.g., cCTA requiring additional discussions on medication and breath-hold instructions), and any additional patient questions, with most interviews lasting 5–7 min.

### Videos

Video I outlines the cardiac CT examination process and provides fundamental background information. It features scenes of entering the hospital, registration, changing rooms, the physician encounter, the actual imaging examination (Fig. 1), and concludes with the patient leaving the hospital and a brief summary of the examination’s risks and benefits. Breath-hold commands are illustrated by an animation. The video is narrated in German, has subtitles and a duration of approximately 3 min.

Video II was limited to the audio and subtitles (Fig. 1) without any visuals. To enhance readability and aesthetics [20] and reduce cognitive load and stress [21], white text on black was chosen. Video II is intended to assess whether an added, simplified explanation of the examination procedure with only text and audio may lead to a reduction in patients’ anxiety and an increase in patients’ satisfaction. In Video II, a black background was chosen for its readability and calming effect.

### Questionnaire

The questionnaire comprises two parts: Part I, administered after the physician encounter and before the examination, and Part II, completed immediately after the examination.

Part I consists of two subsections, first inquiring about socio-demographic information, including age, sex, weight, and medication (Table 1, Appendix 1) using multiple-choice questions. The second subsection is a self-developed questionnaire with 11 items assessing the patient’s perception and well-being before the examination, using visual analog scales (VAS) (Table 2).

**Table 1:** Baseline demographics of the cohort

Patients	Video I (*n* = 67)	Video II (*n* = 69)	Control (*n* = 69)	*p*-value
Age (years)	69.0 [56.5–77.0]	64.0 [57.0–76.0]	70.0 [59.0–78.0]	0.48
Gender				
Female	21 (31.3%)	25 (36.2%)	31 (44.9%)	0.25
Male	46 (68.7%)	44 (63.8%)	38 (55.1%)	
Height (m)	1.72 ± 0.10	1.73 ± 0.10	1.71 ± 0.10	0.50
Weight (kg)	84.0 [73.0–95.0]	78.0 [67.0–94.0]	78.0 [67.0–91.0]	0.14
BMI (kg/m^2^)	28.1 [25.7–32.2]	26.4 [23.8–30.4]	26.1 [23.4–29.9]	0.047*
Level of education				
No degree	2 (3.0%)	0 (0.0%)	1 (1.4%)	0.59
CSE	6 (9.0%)	5 (7.2%)	7 (10.1%)	
GCSE	10 (14.9%)	16 (23.2%)	9 (13.0%)	
A level	2 (3.0%)	3 (4.3%)	0 (0.0%)	
Professional qualification	31 (46.3%)	28 (40.6%)	36 (52.2%)	
Graduate degree	16 (23.9%)	17 (24.6%)	16 (23.2%)	
Place of residence				
City	38 (56.7%)	38 (55.1%)	47 (68.1%)	0.43
Suburb	9 (13.4%)	11 (15.9%)	5 (7.2%)	
Countryside	20 (29.9%)	20 (29.0%)	17 (24.6%)	
Medication		(*n* = 68)		
Beta blocker*** (BB) only	33 (49.3%)	25 (36.8%)	28 (40.6%)	0.65
BB plus Amiodarone or Sotalol	1 (1.5%)	1 (1.5%)	1 (1.4%)	
BB plus Digoxin or Digitoxin	0 (0.0%)	2 (2.9%)	1 (1.4%)	
Verapamil or Diltiazem	1 (1.5%)	0 (0.0%)	2 (2.9%)	
None of the above	32 (47.8%)	40 (58.8%)	37 (53.6%)	
Previous CT/MRI examinations		(*n* = 68)	(*n* = 68)	
Never	9 (13.4%)	7 (10.3%)	8 (11.8%)	0.35
Less than 3 times	27 (40.3%)	34 (50.0%)	38 (55.9%)	
Less than 5 times	21 (31.3%)	15 (22.1%)	11 (16.2%)	
Less than 10 times	5 (7.5%)	10 (14.7%)	9 (13.2%)	
More than 10 times	5 (7.5%)	2 (2.9%)	2 (2.9%)	
Indication				
CT for TAVI planning	29 (43.3%)	19 (27.5%)	25 (36.2%)	0.019**
cCTA	27 (40.3%)	28 (40.6%)	35 (50.7%)	
Valvular thrombosis	1 (1.5%)	4 (5.8%)	0 (0.0%)	
Pre-operative CT only	0 (0.0%)	8 (11.6%)	3 (4.3%)	
Pre-operative CT incl. cCTA	6 (9.0%)	8 (11.6%)	2 (2.9%)	
After HTX	2 (3.0%)	1 (1.4%)	1 (1.4%)	
Other indication	2 (3.0%)	1 (1.4%)	3 (4.3%)	

Values are mean ± standard deviation, median [IQR], or *n* (%). The number of cases per variable is as specified in the table header, unless otherwise stated due to missing values

*BB* beta blocker, *BHC* Bonferroni–Holm correction, *CT* computed tomography, *(G)CSE* (General) Certificate of Secondary Education, *HTX* heart transplantation, *IQR* interquartile range, *MI* myocardial infarction, *MRI* magnetic resonance imaging, *TAVI* transcatheter aortic valve implantation

* No statistical significance in pairwise Dunn tests with Bonferroni–Holm correction (BHC)

** Pairwise Fisher’s exact tests with BHC yield a significant difference in indications CT for TAVI planning and pre-operative CT between study groups I and II (*p* = 0.03)

*** Bisoprolol, Metoprolol or Propranolol

**Table 2:** VAS scores of CT scan-related questions

No.	Questions	Video I	Video II	Control	*p*-value
Information and understanding					
1	I feel well-informed about the upcoming CT examination	9.5 [9.0–9.9] (*n* = 65)	9.5 [9.2–9.8] (*n* = 67)	9.4 [8.8–9.7] (*n* = 68)	0.40
2	The physician consultation was important for my understanding	9.5 [9.0–9.8] (*n* = 65)	9.5 [9.0–9.8] (*n* = 66)	9.5 [8.7–9.7] (*n* = 68)	0.56
3	The informed consent form was important for my understanding	9.0 [8.5–9.8] (*n* = 56)^‡^	9.5 [8.7–9.8] (*n* = 51)^‡^	8.9 [7.1–9.5] (*n* = 46)^‡^	0.02*
4	The video was important for my understanding	9.3 [8.8–9.8] (*n* = 65)	9.1 [8.0–9.8] (*n* = 67)	Not applicable^§^	0.25
Preparation and anxiety before the examination					
5	I learned new details about the upcoming CT examination	9.2 [7.5–9.7] (*n* = 62)	8.8 [4.0–9.7] (*n* = 62)	8.1 [5.0–9.4] (*n* = 59)	0.09
6	The CT examination process was well-explained	9.6 [9.1–9.8] (*n* = 63)	9.5 [9.0–10.0] (*n* = 66)	9.3 [8.8–9.7] (*n* = 68)	0.04**
7	I am satisfied with the amount of information I received	9.6 [9.1–9.8] (*n* = 64)	9.6 [9.1–9.9] (*n* = 67)	9.3 [8.7–9.7] (*n* = 69)	0.02***
8	I feel well-prepared for the upcoming CT examination	9.5 [9.1–9.8] (*n* = 64)	9.5 [9.0–9.8] (*n* = 66)	9.3 [8.5–9.7] (*n* = 69)	0.08
9	My nervousness about the upcoming CT examination is high	2.0 [0.6–5.1] (*n* = 64)	3.4 [0.6–6.1] (*n* = 67)	3.3 [0.5–7.0] (*n* = 69)	0.74
10	I am afraid of the upcoming CT examination	0.6 [0.3–1.6] (*n* = 63)	0.5 [0.2–2.0] (*n* = 66)	0.7 [0.3–2.3] (*n* = 68)	0.59
11	I am afraid of the result	1.3 [0.5–5.0] (*n* = 64)	1.4 [0.3–4.8] (*n* = 66)	1.9 [0.3–6.2] (*n* = 69)	0.59
After the examination/expectation and experience					
12	The CT examination matched my expectations	9.3 [8.9–9.8] (*n* = 64)	9.5 [9.0–9.8] (*n* = 69)	9.2 [8.5–9.7] (*n* = 67)	0.05
13	The CT examination was more pleasant than I imagined	9.2 [7.8–9.7] (*n* = 63)	9.5 [7.1–9.7] (*n* = 69)	9.0 [7.3–9.6] (*n* = 66)	0.37
14	The physician consultation helped reduce my anxiety	9.3 [8.5–9.7] (*n* = 57)	9.5 [8.2–9.8] (*n* = 62)	9.3 [8.5–9.7] (*n* = 56)	0.30
15	The informed consent form helped reduce my anxiety	9.0 [6.9–9.5] (*n* = 46)^‡^	8.8 [5.2–9.7] (*n* = 47)^‡^	8.8 [6.5–9.5] (*n* = 37)^‡^	1.00
16	The video helped reduce my anxiety	9.3 [8.5–9.8] (*n* = 57)	9.2 [5.6–9.7] (*n* = 60)	Not applicable^§^	0.32

VAS scores are separated by study group and presented as median [IQR] with the number of cases in parentheses. Differences in VAS scores between the three study groups are assessed via Kruskal–Wallis omnibus tests and pairwise Dunn post hoc tests with Bonferroni–Holm correction, where applicable. Exceptions are comparisons of two groups (Questions 4 and 16), where Mann–Whitney U tests are performed

*CT* computed tomography, *IC* informed consent, *IQR* interquartile range, *VAS* visual analog scale

* Significant difference between Video II and Control

** No statistical significance in pairwise post hoc tests

*** Significant difference between both Video I and Video II, and Control

^‡^ Only applicable to those patients who studied the IC form

^§^ Patients in Control did not view any video

Part II contains 5 VAS items assessing patients’ experiences during the examination and the impact of the information received during the informed consent process (Table 2).

The VAS measured 10 cm in length: 0 indicated no and 10 cm full agreement.

### Statistics

Categorical variables are reported as count and percentage; continuous variables are reported as mean ± standard deviation or median (interquartile range) for skewed distributions.

Group comparisons were conducted using Pearson’s chi-squared test for categorical variables, analysis of variance for continuous normally distributed variables, or the Kruskal–Wallis H-test for non-normally distributed variables. When statistical significance was reached, post hoc pairwise Fisher’s exact tests or Dunn tests with Bonferroni–Holm correction were performed.

The influence of demographic and anamnestic variables on CT scan-related questions (VAS scores) was tested via multiple linear regression. Because there were significant outliers, robust regression using the Huber estimator was applied [22]. Variables were included in the multivariate models based on significance in univariate analysis and medical expertise conditioned on the absence of multicollinearity. Autocorrelation was ruled out via the Durbin-Watson statistic. Due to heteroscedasticity of residuals, bootstrapping was used to estimate model coefficients and 95% confidence intervals.

To reduce the number of categories for regression analyses, nominal variables were aggregated (Table 3).

**Table 3:** Influence of demographic and anamnestic variables on CT examination-related questions

Q	*n* (I + II + Control)	Model variable	Coeff. [95% CI]	*p*-value	*p*-value overall model
Information and understanding					
1	198 (65 + 66 + 67)	(Intercept)	9.96 [9.52, 10.40]	**< 0.001**	**< 0.001**
		**Age (years)**	−0.01 [−0.02, −0.01]	**< 0.001**	
		**Few or no previous CT/MRI scans**	0.32 [0.04, 0.65]	**0.042**	
2	197 (65 + 65 + 67)	(Intercept)	9.22 [8.88, 9.48]	**< 0.001**	**0.005**
		**Higher education**	−0.40 [−0.78, −0.13]	**< 0.001**	
		Lower education	−0.16 [−0.39, 0.04]	0.15	
		IC form studied	0.17 [−0.08, 0.49]	0.14	
		**Few or no previous CT/MRI scans**	0.20 [0.01, 0.42]	**0.041**	
3	153 (56 + 51 + 46)	(Intercept)	8.71 [8.01, 9.16]	**< 0.001**	**0.002**
		Video I vs. Control	0.43 [−0.02, 1.08]	0.05	
		Video II **vs. Control**	0.56 [0.10, 1.19]	**0.01**	
		**Other pre-existing heart condition**	0.59 [0.18, 1.03]	**0.02**	
		Place of residence: city	−0.33 [−0.71, 0.03]	0.08	
4	132 (65 + 67 + 0)	(Intercept)	9.19 [8.76, 9.48]	**< 0.001**	**0.01**
		**Higher education**	−0.68 [−1.62, −0.17]	**0.004**	
		Lower education	−0.34 [−0.76, 0.01]	0.12	
		Video I vs. Video II	0.29 [−0.06, 0.81]	0.12	
Preparation and anxiety before the examination					
5	181 (62 + 61 + 58)	(Intercept)	6.53 [5.04, 7.65]	**< 0.001**	**0.01**
		**Few or no previous CT/MRI scans**	1.12 [0.18, 2.32]	**0.02**	
		Video I **vs. Control**	1.15 [0.23, 2.17]	**0.03**	
		Video II vs. Control	0.08 [−1.23, 1.20]	0.90	
6	197 (63 + 66 + 68)	(Intercept)	10.00 [9.37, 10.53]	**< 0.001**	**0.002**
		**Age (years)**	−0.01 [−0.02, −0.00]	**0.002**	
		Video I **vs. Control**	0.23 [0.05, 0.43]	**0.02**	
		Video II **vs. Control**	0.23 [0.03, 0.43]	**0.02**	
		IC form studied	−0.15 [−0.37, 0.14]	0.16	
7	200 (64 + 67 + 69)	(Intercept)	9.25 [8.88, 9.53]	**< 0.001**	**0.006**
		Video I **vs. Control**	0.22 [0.03, 0.43]	**0.03**	
		Video II **vs. Control**	0.26 [0.05, 0.47]	**0.008**	
		**IC form received in radiology dept**.	−0.26 [−0.45, −0.01]	**0.03**	
		IC discussion in changing room	0.20 [−0.06, 0.56]	0.15	
8	199 (64 + 66 + 69)	(Intercept)	9.47 [9.14, 9.73]	**< 0.001**	**0.03**
		Video I **vs. Control**	0.24 [0.02, 0.48]	**0.04**	
		Video II vs. Control	0.21 [−0.03, 0.45]	0.06	
		IC form received in radiology dept.	−0.25 [−0.46, 0.02]	0.06	
		Place of residence: city	−0.15 [−0.33, 0.03]	0.11	
9	200 (64 + 67 + 69)	(Intercept)	0.39 [−1.19, 2.10]	0.66	**0.002**
		**Gender**	1.37 [0.34, 2.44]	**0.008**	
		**Few or no previous CT/MRI scans**	0.96 [0.02, 1.94]	**0.048**	
		**Indication: cCTA**	1.66 [0.04, 2.96]	**0.04**	
		Indication: CT for TAVI planning	0.72 [−0.92, 2.08]	0.39	
		Pre-existing hypertension	1.11 [−0.07, 2.23]	0.08	
10	197 (63 + 66 + 68)	(Intercept)	0.09 [−0.86, 0.78]	0.80	**0.02**
		**Indication: cCTA**	0.76 [0.24, 1.80]	**0.007**	
		Indication: CT for TAVI planning	0.25 [−0.16, 0.67]	0.39	
		No pre-existing heart conditions	0.54 [−0.10, 1.56]	0.09	
11	199 (64 + 66 + 69)	(Intercept)	1.01 [−1.49, 3.48]	0.39	**0.005**
		**Indication: CT for TAVI planning**	−1.30 [−2.12, −0.36]	**0.004**	
		**Place of residence: city**	0.89 [0.05, 1.71]	**0.04**	
		BMI (kg/m^2^)	0.06 [−0.02, 0.15]	0.13	
After the examination/expectation and experience					
12	200 (64 + 69 + 67)	(Intercept)	9.08 [8.80, 9.29]	**< 0.001**	**0.007**
		Video I vs. Control	0.18 [−0.07, 0.47]	0.14	
		Video II **vs. Control**	0.27 [0.04, 0.54]	**0.03**	
		**Pre-existing MI**	−0.50 [−1.06, −0.15]	**0.005**	
		Pre-existing VHD	0.15 [−0.05, 0.35]	0.13	
13	198 (63 + 69 + 66)	(Intercept)	7.26 [5.16, 8.73]	**< 0.001**	**0.001**
		**Age (years)**	0.02 [0.00, 0.05]	**0.01**	
		**Higher education**	−0.78 [−1.61, −0.22]	**0.005**	
		Lower education	−0.19 [−0.73, 0.33]	0.47	
		Few or no previous CT/MRI scans	0.36 [−0.11, 0.94]	0.13	
14	172 (57 + 62 + 56)	(Intercept)	9.00 [8.29, 9.39]	**< 0.001**	**0.01**
		**Higher education**	−0.45 [−1.00, −0.08]	**0.01**	
		Lower education	−0.25 [−0.60, 0.08]	0.15	
		Few or no previous CT/MRI scans	0.38 [−0.02, 1.06]	0.06	
15	130 (46 + 47 + 37)*	(Intercept)	4.93 [2.34, 7.41]	**< 0.001**	**0.004**
		**Age (years)**	0.04 [0.01, 0.07]	**0.005**	
		Higher education	−0.89 [−2.07, 0.10]	0.06	
		Lower education	−0.34 [−1.14, 0.45]	0.44	
		**Previous hospitalization/surgery**	1.07 [0.10, 2.90]	**0.045**	
16	117 (57 + 60 + 0)**	(Intercept)	5.67 [2.87, 7.94]	**< 0.001**	**0.002**
		**Age (years)**	0.04 [0.01, 0.07]	**0.006**	
		Higher education	−0.79 [−2.23, 0.06]	0.07	
		Lower education	−0.52 [−1.49, 0.13]	0.21	
		Previous hospitalization/surgery	0.96 [−0.18, 2.96]	0.09	

Relationships were assessed via multiple linear regression. Statistically significant independent variables are in bold. To reduce the number of categories for regression analyses, nominal variables (see Table 1) were aggregated as follows. Level of education: (i) higher education (A level, graduate degree), (ii) lower education (CSE, GCSE), (iii) professional qualification; Previous CT/MRI examinations: (i) few or no previous CT/MRI scans (never, less than 3 times), (ii) several previous CT/MRI scans (greater than or equal to 3 times); Place of residence: (i) city, (ii) outside the city (suburb, countryside); IC discussion: (i) in changing room, (ii) in other locations (in hallway, in medical ward); Indication: (i) CT for TAVI planning, (ii) cCTA (including multiple indications), (iii) other indications (valvular thrombosis, pre-operative CT only, after heart transplantation, and other indication); Previous hospitalization/surgery: (i) yes, (ii) no (including unknown). Note, the age coefficient represents the impact per year of life, therefore being much larger than it may initially appear

*CT* computed tomography, *(G)CSE* (General) Certificate of Secondary Education, *IC* informed consent, *MI* myocardial infarction, *MRI* magnetic resonance imaging, *Q* question, *TAVI* transcatheter aortic valve implantation, *VHD* valvular heart disease

* Only applicable to patients who studied the IC form

** Patients in Control did not view any video

Data curation was performed in Microsoft Excel, version 2010 (Microsoft Corporation). Statistical analyses were conducted with R (v4.4.1, R Foundation for Statistical Computing). All testing was two-tailed, and *p*-values less than 5% were considered to indicate statistical significance.

## Results

### Study population

**Fig. 2:**
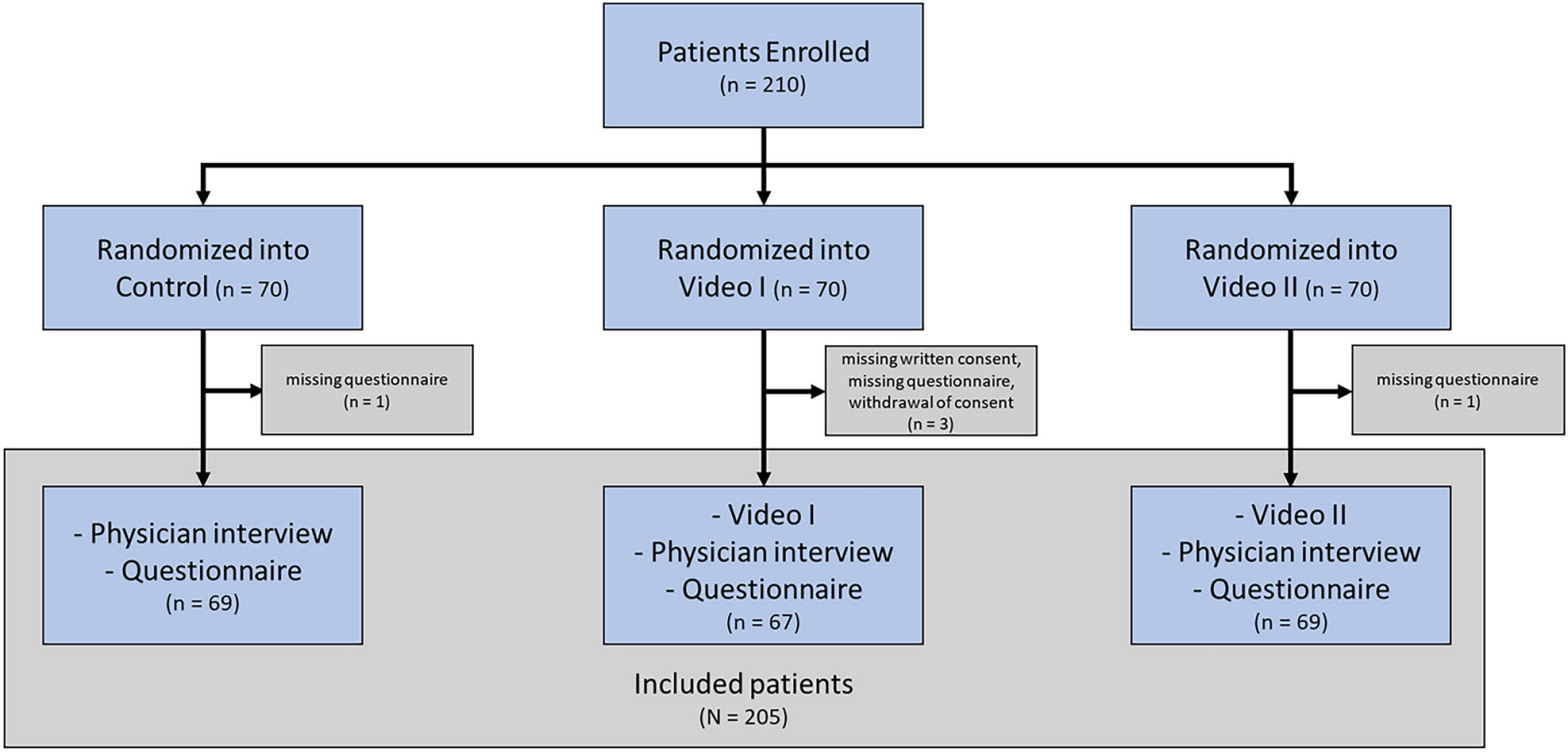
Flowchart of the study population: flowchart detailing the study population from initial screening, through randomization and final patient inclusion

Between January and December 2023, 210 patients were initially enrolled. Five patients were excluded due to incomplete questionnaire, or withdrawn or missing written consent, yielding a final cohort of 205 patients, divided into three groups: Video I (*n* = 67), Video II (*n* = 69), and Control (*n* = 69) (Fig. 2).

Body mass index showed slight differences across groups (Video I: 28.1 [25.7–32.2] kg/m²; Video II: 26.4 [23.8–30.4] kg/m²; Control: 26.1 [23.4–29.9] kg/m²; *p* = 0.047), though pairwise post hoc tests showed no significant differences between groups. All other baseline characteristics were homogeneous across groups, including weight and height (Table 1, Appendix 1).

Indication for cardiac CT varied significantly among groups (*p* = 0.02), with pairwise post hoc tests showing significant differences between Video I and Video II for TAVI planning and pre-operative CT indications (*p* = 0.03). No further significant differences were observed in demographic or clinical variables (Table 1, Appendix 1).

### Questionnaire assessment

In total, 63.4% of patients completed all questions of the questionnaire, including questions regarding baseline demographics and 16 VAS items concerning the patient’s feelings, well-being and experience.

Overall, 75.7% of patients reviewed the informed consent form, with significant group differences (Video I: 86.2%; Video II: 75.0%; Control: 66.7%; *p* = 0.03), with pairwise post hoc comparison revealing a significant difference between Video I and the Control (*p* = 0.03).

#### Information and understanding

**Fig. 3:**
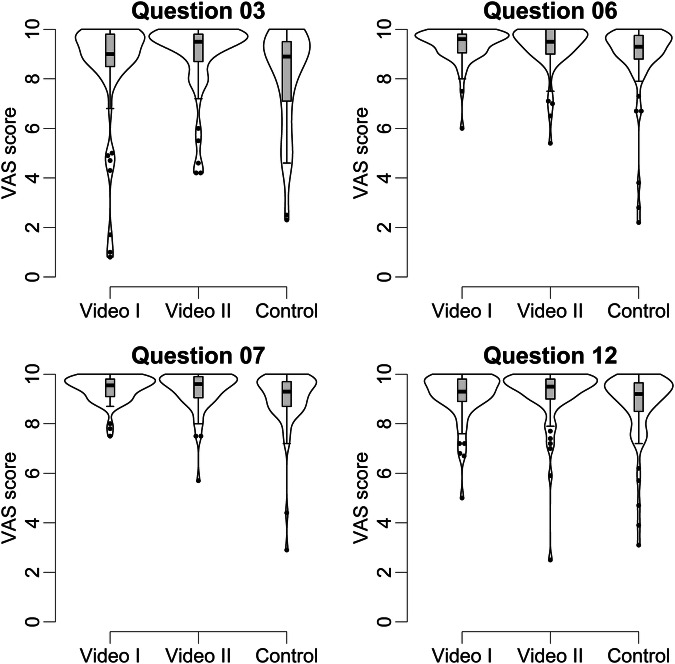
Overlay of box and violin plots: visualization of VAS scores for questions 3, 6, 7 and 12 with box plots overlaid with violin plots. This representation shows that patients from Videos I and II generally reported higher VAS scores compared to the Control, even though median values are similar. VAS, visual analog scale

VAS scores for Q3, assessing the informed consent form’s role in patient understanding the upcoming CT, varied significantly (Video I: 9.0 [8.5–9.8], Video II: 9.5 [8.7–9.8], Control: 8.9 [7.1–9.5]; *p* = 0.02; Fig. 3). Post hoc tests showed this to be attributed to higher scores in Video II compared to the Control.

No statistically significant differences were observed in responses to Q1, Q2, and Q4 across the study groups (Table 2).

#### Preparation and anxiety before the examination

Significant differences among groups were noted for Q6, evaluating patients’ clarity about the examination process (Video I: 9.6 [9.1–9.8], Video II: 9.5 [9.0–10.0], Control: 9.3 [8.8–9.7]; *p* = 0.04; Fig. 3), though pairwise post hoc tests did not show significant differences between individual groups.

Q7, measuring satisfaction with the information provided, showed statistically significant group differences (Video I: 9.6 [9.1–9.8], Video II: 9.6 [9.1–9.9], Control: 9.3 [8.7–9.7]; *p* = 0.02; Fig. 3) with significantly higher scores in both Video I and Video II compared to the Control. Q8, related to patients’ sense of preparedness, showed a trend toward higher VAS scores in Groups I and II, but did not reach statistical significance (Video I: 9.5 [9.1–9.8], Video II: 9.5 [9.0–9.8], Control: 9.3 [8.5–9.7]; *p* = 0.08). No statistically significant differences were found in responses to Q5 and Q9-11 (Table 2).

#### After the examination/expectation and experience

For Q12, assessing correspondence of the CT examination to participants’ expectations, Groups I and II trended toward higher VAS scores, though this did not reach statistical significance (Video I: 9.3 [8.9–9.8]; Video II: 9.5 [9.0–9.8], Control: 9.2 [8.5–9.7]; *p* = 0.05; Fig. 3). No significant differences were found in responses to Q13-16 (Table 2).

### Influence of demographic and anamnestic variables

**Fig. 4:**
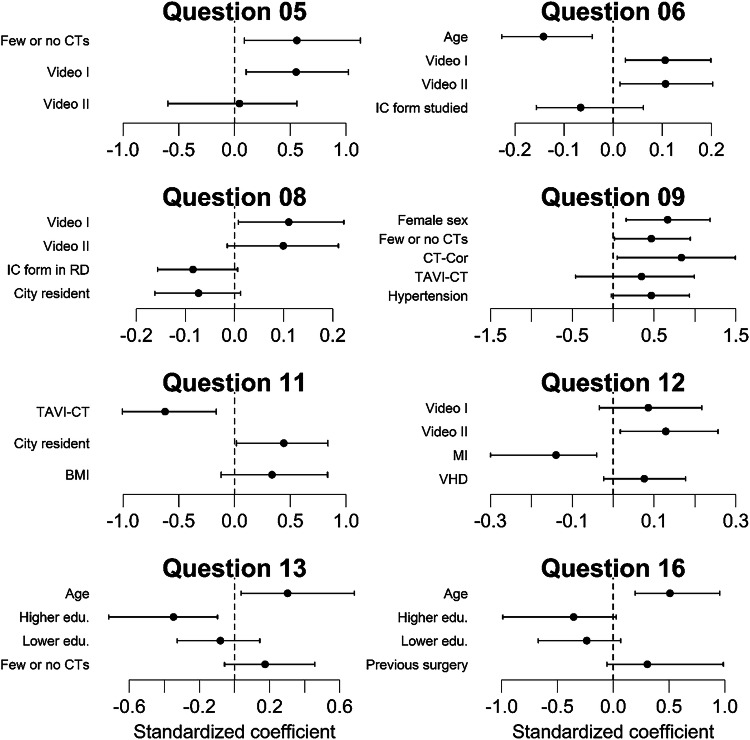
Plots of standardized linear regression model coefficients: visualization of patient characteristics’ effects on responses to selected questions (see Table 3). The distance of the coefficient estimate (small filled circles) from the dashed zero-line indicates the effect size, with whiskers showing the 95% CI. Predictors are considered statistically significant if the 95% CI does not intersect the zero-line. Horizontal axes are scaled individually for clarity. CI, confidence interval; CT, computed tomography; IC form, informed consent form; RD, radiology department; edu., education; BMI, body mass index; MI, myocardial infarction; TAVI, transcatheter aortic valve implantation; VHD, valvular heart disease

A statistically significant multivariate linear regression model to identify factors predicting the response behavior could be constructed for each VAS item (*p* < 0.03). In these models, two or more of the following baseline demographics were found to be partially explanatory to the response and independently statistically significant: age, gender, level of education, previous experience with CT/MRI or hospitalization, indication, previous cardiac conditions, place of residence, location where the informed consent form was handed out, and group allocation (Table 3, Fig. 4). Additionally, several demographic variables had an influence on the response behavior, but did not reach statistical significance when individually tested (Table 3).

#### Age

Age significantly predicted responses to Q1, Q6, Q13, Q15 and Q16, explaining between −0.01 and 0.04 of the VAS score per year of life (*p* ≤ 0.01). Increased age was associated with feeling less well-informed (Q1: −0.01; *p* < 0.001), perceiving the examination process as less well-explained (Q6: −0.01; *p* = 0.002), experiencing the examination as more pleasant than expected (Q13: 0.02; *p* = 0.01), and rating both the informed consent form (Q15: 0.04, *p* = 0.005) and video (Q16: 0.04; *p* = 0.006) as more helpful to reduce one’s anxiety.

#### Examination experience

Patients with few or no previous CT/MRI reported feeling more informed and nervous about the examination than patients with more experience, with a significant impact on Q1, Q2, Q5 and Q9, increasing VAS scores by a median of 0.20–1.12 (*p* < 0.05). Specific associations included feeling well-informed (Q1: 0.32; *p* = 0.04), deeming the physician encounter important for understanding (Q2: 0.20; *p* = 0.04), learning new details (Q5: 1.12; *p* = 0.02), and being nervous about the upcoming examination (Q9: 0.96; *p* = 0.048).

#### Education

Education level significantly impacted responses to Q2, Q4, Q13 and Q14, with VAS scores decreasing by a median of −0.78 to −0.40 in patients with higher education (*p* ≤ 0.01). Such patients found the physician encounter (Q2: −0.40; *p* ≤ 0.001) and the video (Q4: −0.68; *p* = 0.004) less important for understanding, rated the examination experience as less pleasant than expected (Q13: −0.78; *p* = 0.005), and found the physician encounter less helpful in reducing anxiety (Q14: −0.45; *p* = 0.01) compared to less educated patients.

#### Group allocation

Group allocation significantly influenced responses to Q3, Q5, Q6, Q7, Q8 and Q12, explaining median VAS score differences between 0.22 and 1.15 (*p* ≤ 0.04). Patients in group Video I (vs. Control) reported having learned more new details (Q5: 1.15; *p* = 0.03), finding the examination process well-explained (Q6: 0.23; *p* = 0.02), being more satisfied with the amount of information received (Q7: 0.22; *p* = 0.03), and feeling well-prepared for the upcoming CT examination (Q8: 0.24; *p* = 0.04). Patients in group Video II (vs. Control) perceived the informed consent form as more important (Q3: 0.56; *p* = 0.02), found the examination process well-explained (Q6: 0.23; *p* = 0.02), were satisfied with the amount of information (Q7: 0.26; *p* = 0.008), and experienced better alignment between their expectations and the actual examination (Q12: 0.27; *p* = 0.03).

#### Gender

Female patients reported significantly higher levels of nervousness about the upcoming CT examination (Q9: 1.37; *p* = 0.008).

#### Indication

CT indication (e.g., cCTA or TAVI planning) significantly predicted responses to Q9, Q10 and Q11, with median changes in VAS score ranging from −1.30 to 1.66 (*p* ≤ 0.04). Patients undergoing cCTA reported higher levels of nervousness (Q9: 1.66; *p* = 0.04) and anxiety about the CT examination (Q10: 0.76; *p* = 0.007). TAVI planning was associated with reduced anxiety about the results of the examination (Q11: −1.30; *p* = 0.004).

#### Other baseline demographics

While occasionally statistically significant, the influence of the following baseline variables was comparatively small: other pre-existing heart condition; pre-existing myocardial infarction; location where the informed consent form was received; place of residence; previous hospitalization/surgery (Table 3).

## Discussion

This study found that VAIC before cardiac CT was positively received and enhanced patient satisfaction and understanding of the examination process. Noteworthy, several baseline demographics could predict patients’ perceptions of the examination process.

As in other studies, showing a video in preparation of an imaging examination has a measurable, positive effect on patients’ perception of the examination process [17, 18]. Part of this positive effect can also be achieved through a minimal video (Video II, black screen with audio and subtitles), while other effects can only be achieved by an instructional video (Video I) with visual elements that illustrate, for example, the examination process using video and animations (Table 2, Fig. 3).

In addition, there are intrinsic patient characteristics influencing the individual patient’s perception and assessment of the examination situation, e.g., age [23], educational background [24], previous comparable examinations (CT/MRI) [25], indication for the examination (likely functioning as surrogate for patients’ expectance) [26], gender [9, 24, 26, 27], and place of residence, all of which have also been shown to be predictive in our study in descending order of importance (Table 3).

Providing sufficient information before any examination is essential. As in previous studies, the physician encounter was rated as the most important component of preparation [28], suggesting that video content cannot completely replace a personal consultation, likely because the physician interactions can be tailored to patients’ individual needs and circumstances [23, 28, 29]. Consistent with our findings, prior medical experience [23] and education level [28, 29] influenced the perceived importance of physician consultations. Patients with less previous experience often find personal interaction more beneficial, while those with higher educational backgrounds may view it as less important, likely because they can read and comprehend the informed consent (IC) form more easily, leaving fewer questions.

For patients who viewed Video II, written information gained importance, suggesting that while Video II familiarized the patient with the examination process, it was less effective in providing the necessary information. This may have prompted the patient to turn to the IC form and read it more thoroughly than a naïve patient (Control) would have done. Conversely, Video I may have raised similar questions, but was able to answer them through the additional visual components.

Ultimately, for patients who viewed the instructional video (Video I), the video emerged as a more important source of information than the IC form. This suggests that VAICICI improves understanding of informed consent, especially given the differences in reading ability depending on educational levels and stress levels [23]. This also explains why patients with higher education rated the video as less essential. Videos can present information in a standardized and simplified form [30], potentially better accommodating patients’ varying reading abilities, mental states, or unique circumstances compared to printed materials or a clinician’s verbal explanation alone [23]. These findings highlight the need for high-quality, accessible, and comprehensible video material.

Although not directly assessed in this study, perceiving a video as informative does not necessarily translate into improved understanding or compliance. However, a well-perceived video may help reduce anxiety. While combining well-structured information with personal communication is generally considered the most effective solution for anxiety reduction [31], our results did not conclusively demonstrate this, likely because anxiety levels were already minimal.

Patients with less CT experience and those who viewed Video I were more likely to report learning new details. Previous CT/MRI experience significantly influenced patient responses. Patients with ‘few or no previous scans’ felt more informed and learned more new details, but reported higher nervousness compared to those with more experience (Table 3). These findings suggest that less experienced patients benefit more from VAIC in terms of education but may require additional support to alleviate anxiety. Tailoring patient education and reassurance to individual familiarity with medical imaging procedures is essential for optimizing the informed consent process. The video format provided a comprehensive explanation of the examination process, with patient assessment not differing between Videos I and II. However, the clarity of the examination explanation was rated worse with increasing age, potentially due to reduced comprehension capacity [23]. Nevertheless, patients who viewed any video expressed greater satisfaction with the amount of information provided, likely because the videos were concise, focusing on key details, making them more accessible than the written information in the IC form. Additionally, patients who viewed a video reported feeling better prepared, although this feeling of preparedness was not objectively verified, such as through testing.

The videos had no measurable impact on patients’ nervousness levels. Only older patients reported that the video was helpful in reducing their anxiety. As in other studies, women reported higher levels of nervousness [9, 24, 26, 27]. Anxiety levels also varied depending on the CT indication; patients undergoing cCTA [27] and those without prior CT examinations [9] exhibited higher levels of nervousness and anxiety. This is likely due to unfamiliarity with the examination and concerns about its outcome. Conversely, patients undergoing CT for TAVI planning demonstrated reduced anxiety, potentially due to their familiarity with the planned procedure and the comparatively low clinical significance of the results since valvular heart disease had already been diagnosed [9]. Although anxiety is influenced by multiple factors [9, 27], identifying specific underlying causes was beyond the scope of this study and could not be explored further, as baseline anxiety levels in our study population were very low.

Implementing VAICICI resulted in better alignment between patients’ expectations and their actual experiences. Although the information provided by the video was initially not considered very important, patients found it more helpful after the examination. The video clarified elements such as the duration of the examination—often confused with the much longer duration of an MRI—and breathing commands, helping to set accurate expectations. Interestingly, in contrast to age and educational background, group allocation had no influence on how (un)pleasant patients found the examination. Perceived pleasantness increased with age, potentially due to a mismatch between expectations and the relatively comfortable examination experience. Conversely, patients with higher education tended to find the examination less pleasant, suggesting a different or more critical point of reference.

Although personal contact is generally considered the most effective way to reduce anxiety, patients with higher education rated the physician’s consultation as less important. Older patients, on the other hand, rated the IC form and video better. Since no significant differences in anxiety were observed between Videos I and II, it may be concluded that having the text read aloud offers advantages over the standard informed consent process alone. While a video contributes positively to patients’ understanding, it cannot replace the patient-doctor interaction, whether for care or medico-legal reasons.

Consistent with other studies, the video improved patients’ feelings of being well-informed and prepared, but had no measurable effect on anxiety [16]. Clearly, patient understanding and nervousness may influence compliance and physiological parameters like heart rate. However, the impact of VAICICI on image quality remains to be investigated.

The VAICICI trial highlights the value of video-assisted informed consent in cardiac imaging by offering a standardized and accessible medium to convey complex information. Videos cater to diverse learning preferences and reduce the cognitive burden associated with traditional consent methods. By improving patient satisfaction and understanding, VAICICI bridges communication gaps, particularly for patients with less prior experience or differing educational backgrounds. These findings support integrating audiovisual aids into routine clinical practice to enhance patient-centered care. Future research should investigate VAICICI’s impact on compliance, physiological parameters, and imaging outcomes to reinforce its role in contemporary informed consent processes.

### Limitations

This study has limitations inherent to its single-center design, potentially reducing the diversity of the patient population. Because the examination was performed at a high-volume specialized center, the setting may have impacted patient experiences, as such facilities may be perceived as more stressful than outpatient facilities or smaller hospitals. The patient population was also skewed by a high proportion of older patients with prior imaging experience, limiting our ability to analyze the specific responses of CT-naïve patients.

Additionally, patient assignment to inpatient or outpatient status, determined by secondary referral, introduced variability in examination types. Despite efforts to achieve a balanced group distribution, these factors may limit the generalizability of our findings.

This study relied on validated but self-reported measures, namely VAS scores. While subjective, these are validated tools commonly used to assess patient experiences. Future research could complement them with objective measures, e.g., validated behavioral tests, to enhance generalizability.

While all staff members were trained in the informed consent procedures, interpersonal communication skills may vary. This variability in communication style and engagement could have influenced patient responses and is a study limitation. Furthermore, response biases, including acquiescence bias, courtesy bias, demand characteristics, and social desirability bias, may have influenced the self-reported outcomes in this study. While the use of anonymous, self-administered visual analog scales was intended to mitigate these effects, they remain a potential limitation. Future research could incorporate alternative approaches, such as mixed-methods designs or objective measures focusing on imaging outcomes, to validate and complement patient-reported outcomes.

Baseline anxiety levels in our study population were very low, with median VAS scores of 0.7 [0.3–2.3] in the control group (Table 2) prior to cardiac CT. Consequently, significant further reductions in anxiety were unlikely. Nevertheless, the primary goals of improving patient satisfaction and understanding were achieved.

Variations in preparatory protocols, such as the administration of beta-blockers or vasodilators during cardiac CT, could influence patient experiences and thus self-reported outcomes. Although these medications were not a significant factor in our study due to their specific and limited use, we acknowledge this as a potential source of bias. Future research should investigate the effects of protocol differences, including medication use, on both patient experience and imaging outcomes in larger and more diverse cohorts.

## Conclusion

VAICICI improves patient satisfaction and understanding of the cardiac CT examination, with little variation in effectiveness across baseline demographics, suggesting it is a valuable addition to the informed consent process. Although no significant reduction in anxiety was observed, the potential impact of VAICICI on compliance and image quality warrants further investigation.

## Supplementary information

ELECTRONIC SUPPLEMENTARY MATERIAL

## Abbreviations

cCTA: Coronary CT angiography
CT: Computed tomography
IC: Informed consent
TAVI: Transcatheter aortic valve implantation
VAIC: Video-assisted informed consent
VAICICI: Video-assisted informed consent in cardiac imaging
VAS: Visual analogue scales
