# Supplementary material for: Video-assisted informed consent in cardiac imaging: influence on patient experience during CT—the randomized controlled VAICICI Trial
Source: Eur Radiol. 2025 Jun 27;36(1):148–62. doi: 10.1007/s00330-025-11741-8 (PMC12711976; doi:10.1007/s00330-025-11741-8)
Supplement: Supplementary file 1 — ELECTRONIC SUPPLEMENTARY MATERIAL [file 330_2025_11741_MOESM1_ESM.pdf]

Video-assisted informed consent in cardiac imaging: influence on patient experience during CT – the randomized controlled VAICICI-Trial

Electronic Supplementary Material (ESM)

Eur Radiol (2025) Gohmann RF, Mettke SM, Krieghoff C et al.

Patients	Video I (n=67)	Video II (n=69)	Control (n=69)	*p*
Medical devices			(n=68)	0.99
glasses only	49 (73.1%)	52 (75.4%)	49 (72.1%)	
hearing aid only	1 (1.5%)	0 (0.0%)	1 (1.5%)	
glasses and hearing aid	9 (13.4%)	8 (11.6%)	8 (11.8%)	
none of the above	8 (11.9%)	9 (13.0%)	10 (14.7%)	
Pre-existing psychiatric conditions				0.95
anxiety disorder	2 (3.0%)	2 (2.9%)	1 (1.4%)	
depression	1 (1.5%)	1 (1.4%)	3 (4.3%)	
other diagnosis	1 (1.5%)	1 (1.4%)	1 (1.4%)	
multiple diagnoses	2 (3.0%)	1 (1.4%)	0 (0.0%)	
unknown to patient	2 (3.0%)	1 (1.4%)	1 (1.4%)	
none of the above	59 (88.1%)	63 (91.3%)	63 (91.3%)	
Date of last CT/MRI examination				0.60
never	9 (13.4%)	7 (10.1%)	8 (11.6%)	
less than 3 months	14 (20.9%)	13 (18.8%)	11 (15.9%)	
less than 1 year	13 (19.4%)	15 (21.7%)	8 (11.6%)	
more than 1 year	31 (46.3%)	34 (49.3%)	42 (60.9%)	
Previous hospitalization/surgery				0.98
yes	60 (89.6%)	61 (88.4%)	61 (88.4%)	
no	5 (7.5%)	6 (8.7%)	7 (10.1%)	
unknown to patient	2 (3.0%)	2 (2.9%)	1 (1.4%)	
Pre-existing CHD				0.91
yes	28 (41.8%)	28 (40.6%)	26 (37.7%)	
no	39 (58.2%)	41 (59.4%)	43 (62.3%)	
Pre-existing MI				0.29
yes	9 (13.4%)	5 (7.2%)	4 (5.8%)	
no	58 (86.6%)	64 (92.8%)	65 (94.2%)	
Pre-existing HF				0.90
yes	26 (38.8%)	25 (36.2%)	24 (34.8%)	
no	41 (61.2%)	44 (63.8%)	45 (65.2%)	
Pre-existing VHD				0.40
yes	37 (55.2%)	35 (50.7%)	30 (43.5%)	
no	30 (44.8%)	34 (49.3%)	39 (56.5%)	
Pre-existing hypertension				0.37
yes	51 (76.1%)	50 (72.5%)	57 (82.6%)	
no	16 (23.9%)	19 (27.5%)	12 (17.4%)	
Other pre-existing heart condition				0.89
yes	7 (10.4%)	9 (13.0%)	7 (10.1%)	
no	60 (89.6%)	60 (87.0%)	62 (89.9%)	
No pre-existing heart conditions				0.99
yes	7 (10.4%)	7 (10.1%)	8 (11.6%)	
no	60 (89.6%)	62 (89.9%)	61 (88.4%)	
Informed consent discussion	(n=61)	(n=66)		0.21
in hallway	4 (6.6%)	2 (3.0%)	7 (10.1%)	
in changing room	57 (93.4%)	61 (92.4%)	59 (85.5%)	
in medical ward	0 (0.0%)	3 (4.5%)	3 (4.3%)	
Informed consent form received	(n=62)	(n=67)		0.74
in medical ward	7 (11.3%)	10 (14.9%)	11 (15.9%)	
in radiology dept.	55 (88.7%)	57 (85.1%)	58 (84.1%)	

Appendix 1: Additional baseline demographics of the cohort. Values are given as absolute numbers and (percent). The number of cases per variable is as specified in the table header, unless otherwise stated due to missing values.
CT = computed tomography; MRI = magnetic resonance imaging; CHD = coronary heart disease; HF = heart failure; MI = myocardial infarction; VHD = valvular heart disease.
